# Multi-strategy genome-wide association studies identify the DCAF16-NCAPG region as a susceptibility locus for average daily gain in cattle

**DOI:** 10.1038/srep38073

**Published:** 2016-11-28

**Authors:** Wengang Zhang, Junya Li, Yong Guo, Lupei Zhang, Lingyang Xu, Xue Gao, Bo Zhu, Huijiang Gao, Hemin Ni, Yan Chen

**Affiliations:** 1 Cattle Genetics and Breeding Group, Institute of Animal Science (IAS), Chinese Academy of Agricultural Sciences (CAAS), Beijing 100193, China; 2 Animal Science and Technology College, Beijing University of Agriculture (BUA), Beijing 102206, China

## Abstract

Average daily gain (ADG) is the most economically important trait in beef cattle industry. Using genome-wide association study (GWAS) approaches, previous studies have identified several causal variants within the *PLAG1, NCAPG* and *LCORL* genes for ADG in cattle. Multi-strategy GWASs were implemented in this study to improve detection and to explore the causal genes and regions. In this study, we conducted GWASs based on the genotypes of 1,173 Simmental cattle. In the SNP-based GWAS, the most significant SNPs (rs109303784 and rs110058857, P = 1.78 × 10^−7^) were identified in the *NCAPG* intron on BTA6 and explained 4.01% of the phenotypic variance, and the independent and significant SNP (rs110406669, P = 5.18 × 10^−6^) explained 3.32% of the phenotypic variance. Similarly, in the haplotype-based GWAS, the most significant haplotype block, Hap-6-N1416 (P = 2.56 × 10^−8^), spanned 12.7 kb on BTA6 and explained 4.85% of the phenotypic variance. Also, in the gene-based GWAS, seven significant genes were obtained which included *DCAF16* and *NCAPG*. Moreover, analysis of the transcript levels confirmed that transcripts abundance of *NCAPG* (P = 0.046) and *DCAF16* (P = 0.046) were significantly correlated with the ADG trait. Overall, our results from the multi-strategy GWASs revealed the *DCAF16-NCAPG* region to be a susceptibility locus for ADG in cattle.

With the recent emergence of genome-wide association studies (GWASs)^1^, major advances have been made in the understanding and practice of functional gene discovery and quantitative trait locus (QTL) mapping^2^,^3^,^4^. Although the Single Nucleotide Polymorphism (SNP)-based GWAS has been useful for identifying causal variants^5^, this strategy has its limitations. This approach overlooks the interaction between SNPs within a gene, misses weak signals that aggregate within related SNP sets, and incurs a severe penalty for multiple testing^6^.

To increase the statistical power and limit the false discovery rate (FDR) associated with GWAS analyses, GWASs have been improved using haplotype-based^7^,^8^,^9^,^10^ and gene-based^11^,^12^,^13^,^14^ strategies to assess complex and quantitative traits in human and domestic animals. The haplotype-based GWAS has high statistical power^15^,^16^ and aims to identify causal haplotypes with specific combinations^7^. Haplotype-based GWASs have recently identified susceptibility haplotypes or blocks for coronary artery disease^7^, low-density lipoprotein cholesterol^8^, triglyceride levels^9^, and boar taint^10^.

Because the gene-based GWAS analysis involves all variants within a gene, it has reduced the number of required tests and is more powerful than the simple SNP-based GWAS^17^,^18^,^19^. Several gene-based GWAS methods have been developed, including the genetic similarity gene-based GWAS^20^, entropy-based joint analysis^21^, and extended Simes procedure association analysis^17^. Risk genes have been successfully identified for several human diseases, including multiple sclerosis^11^, hypertension^13^, and Alzheimer’s disease^14^. However, there has been little gene-based GWAS research on quantitative traits in domestic animals.

In the beef cattle industry, average daily gain (ADG) is an economically important growth trait that contributes to the production efficiency and economic benefits of graziery. Table 1 lists the ADG-associated QTL positions and candidate genes that have been reported in cattle. Notably, *PLAG1* and *NCAPG*-*LCORL*, known loci that are linked to adult human height^22^,^23^,^24^, have been associated with growth traits and body size in cattle^25^,^26^,^27^,^28^,^29^. The dissection of a QTL and the fine mapping of QTNs involved in bovine stature have been reported for the *PLAG1* gene^28^. The mechanism of the effect of *PLAG1* on growth and fertility has been clearly illustrated, and *PLAG1* knockout mice have highlighted the importance of *PLAG1* in postnatal growth and reproduction^30^.

Using 1,173 samples genotyped by Illumina BovineHD Beadchip, multi-strategy GWASs were performed to explore candidate genes or QTL regions for the ADG trait in Simmental cattle. Transcripts abundance of candidate genes were also examined and validated to be associated with ADG trait in this study. Identification of the promising candidate genes for further studies will greatly dissect the molecular mechanisms underlying ADG trait in cattle and has the practical in breeding program for the improvement of carcass weight in breeding program.

## Materials and Methods

### Ethics statement

All animal procedures were conducted in strict accordance with the guidelines proposed by the Chinese Council on Animal Care, and all protocols were approved by the Science Research Department of the Institute of Animal Science, Chinese Academic of Agriculture Sciences (Beijing, China). The use of animals and private land in this study was approved by their respective owners.

### Phenotype Data

The resource population consisted of 1,173 Simmental cattle that were born between 2008 and 2013 in Ulagai, Inner Mongolia. After weaning, all calves were transferred to a fattening farm in Beijing and fattened in the same pens for 8–12 months. All cattle were fed with identical feed, which consisted of silage, brewer’s grain, bean dregs, breadcrumbs, and maize. We measured each bull’s body weight at the following five time points: birth, upon entering the fattening farm, 12 months of age, 18 months of age, and before slaughter. The growth curve analyses closely followed the linear regression during the fattening period (see Supplementary Fig. S1), and the slope of the regression line therefore represented the average daily gain (ADG) during the fattening period.

### Genotype Data

The genotypes of the 1,173 beef cattle were obtained by Illumina BovineHD BeadChip, which included 774,660 SNPs. Quality control procedures were carried out using PLINK 1.7 software^31^ to remove SNPs with a call rate less than 95%, a minor allele frequency (MAF) less than 0.05 and a significant deviation from the Hardy-Weinberg equilibrium (P < 10^−5^); moreover, animals with more than 10% missing genotypes were removed from the dataset. Missing alleles were imputed using Beagle 4.1 software^32^ to guarantee the accuracy and effectiveness of the statistics^33^,^34^.

### Gene Annotation

A total of 24,596 genes were downloaded from the Ensembl Genes database (http://www.ensembl.org/index.html, UMD3.1), including the coding and non-coding RNA. To address the regulatory regions and linkage disequilibrium in SNPs^18^,^35^, we defined the gene boundary as ±50 kb upstream and downstream of the gene. Each gene was covered by three or more SNPs in the genotyping BeadChip, and 23,856 genes remained to be analyzed.

### SNP-based GWAS

A standard MLM for GWAS was performed by extending the Henderson notation as follows:


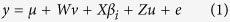


where *y* represented a vector of ADG, *μ* represented the population mean, *v* represented a vector of fixed effects, *β*_*i*_ denoted the effect of the *i*th SNP, *u* represented a vector of the polygenic effects and *e* represented the residual. *W, X* and *Z* represented the incidence matrices for *v, β*_*i*_ and *u. Z* was the genetic additive matrix constructed by SNPs, termed as *kinship*. As described by Lopes^36^, we built kinship using 50,000 random SNPs across autosomes. In this model, we considered sex, birth year, calving season and population stratification as fixed effects.

The percent phenotypic variance that was explained by a single significant SNP was calculated as follows:


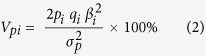


where *p*_*i*_ and *q*_*i*_ represented the allele frequencies for the *i*th SNP, *β*_*i*_ denoted the effect of the *i*th SNP, and *σ*_*p*_^2^ represented the phenotypic variance. The R package *heritability* (https://cran.r-project.org/web/packages/heritability/index.html) was used to estimate the ADG-associated heritability and genetic variance.

### Haplotype-based GWAS

Haplotype-based GWAS was performed using the method proposed by Gregersen VR *et al*.^10^. Haplotype blocks were established based on pairwise measures of the linkage disequilibrium (LD)^37^ and implemented using the PLINK 1.7 software with a block window that was less than 100 kb. The haplotype block estimation option was --blocks --ld–window-kb 100. After the haplotype block partitioning, haplotypes for each sample were calculated using a standard expectation-maximum (EM) algorithm, and the program was conducted using the R package *haplo.stats* (URL: https://cran.r-project.org/web/packages/haplo.stats/index.html). Haplotype association analyses were implemented in the R package *lme4* (URL: https://cran.r-project.org/web/packages/lme4/index.html) using the MLM equation as follows:


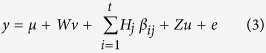


where *y* represented a vector of ADG, *μ* represented the population mean, *v* represented a vector of fixed effects, *β*_*ij*_ represented the effect of the *i*th haplotype in the *j*th block (which contained t haplotypes), *u* denoted polygenic effects for each individual, and *e* represented the residual. *W, H*_*i*_ and *Z* were the incidence matrices for *v, β*_*ij*_, and *u*. A *Chi-square* hypothesis test with *df* = 1 was used to calculate the significance level of the haplotype block as follows:


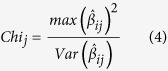


w*here* 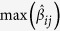 denoted the maximum effective haplotype at the *j*th block, and 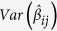 represented the variance of 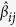 obtained via mixed model equations.

The percent phenotypic variance (*V*_*pj*_) explained by the *j*th block was calculated using a two-step approach. Firstly, the effect of haplotypes at the *j*th block was estimated using least square (LS) method and all *j*th block haplotypes were clustered into two groups (G1 and G2) based on the estimated effects. Each sample was defined as 0, 1, and 2 (G1/G1, G1/G2 and G2/G2) according to the EM results. We then calculated *V*_*pj*_ as follows:


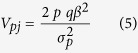


where *β* represented the regression coefficient of the phenotype on the indicator (0, 1 and 2), and *p* and *q* indicated the frequencies of G1 and G2, respectively.

### Gene-based GWAS

We conducted a gene-based GWAS method using a principal component analysis (PCA) according to the method proposed by Kai Wang *et al*.^38^. First, principle components (PCs) were constructed based on an intragenic SNP indicator, and we selected the PCs based on a cumulative contributed proportion >85%. Second, the estimate breeding value (EBV) was calculated based on genomic best linear prediction (GBLUP) with fixed effects (sex, birth year, calving season and population stratification) and random effects (polygenic effects). Third, the effectiveness of each PC and statistical hypothesis test was calculated. The general linear model was:


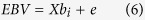


where *b*_*i*_ represented the regression coefficient of the phenotype on the PC, *X* represented the vector of the PC, and *e* represented the residual. The following *Chi-square* hypothesis testing (*df* = 1) formula was used:


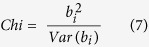


For each gene, we selected the minimum P-value for the PCs when the PC number exceeded two. The significant threshold was set based on the permutation testing to overcome false positive discovery^39^. Thus, 1,000 permutation cycles were performed (23,856,000 multiple tests), and the 240,000^th^ highest value represented the cut-off point for the 1% level of significance.

### Gene expression level

To validate whether the explored gene resulting from the three GWAS methods was associated with ADG trait, transcript abundance in longissimus dorsi muscle tissue was measured. We selected 28 steers randomly in 2014. Longissimus dorsi muscle samples were collected from steers at slaughter and stored in liquid nitrogen. Total RNA was isolated using the TRIzol Reagent total RNA extraction kit (Invitrogen, Carlsbad, CA, USA) and precipitated with ethanol.

Primers were designed using the Primer 5 software and were approximately 200 bp in length (Supplementary Table S1). Real-time PCR was performed to examine the expression level of selected genes using the SYBR^®^ Fast qPCR Mix (Takara Bio, Otsu, Japan) with the Applied Biosystems^®^ 7500 Real-Time PCR Systems (Applied Biosystems, Foster City, CA, USA). Expression values were normalized to *GAPDH* as the internal control. The mean fold change in expression of the target genes was calculated using the 2^−ΔΔCt^ method.

Correlation analyses were conducted using R version 3.2.2 (https://cran.r-project.org, 18/3/2016). Correlations were derived for all candidate genes expression and phenotypic data with 28 random steers from the same year. General linear model (GLM) was used and the fixed effects included calving season and population stratification effects.

## Results and Discussion

### Phenotype description and genetic parameters

The phenotypic distribution followed a Gaussian distribution with a mean of 0.98 kg/day, a maximum of 1.87 kg/day, a minimum of 0.54 kg/day, and a standard deviation (SD) of 0.16 kg/day. The heritability (h^2^) of the average daily gain (ADG) was 0.48, with an additive genetic variance (Va) equal to 0.012.

Following the quality control and imputation, 1,141 samples with 669,742 SNPs remained. Cleaned SNPs were uniformly distributed over the whole bovine genome with a mean inter-marker space of 4.52 kb.

### SNP-based GWAS results

**Figure 1:**
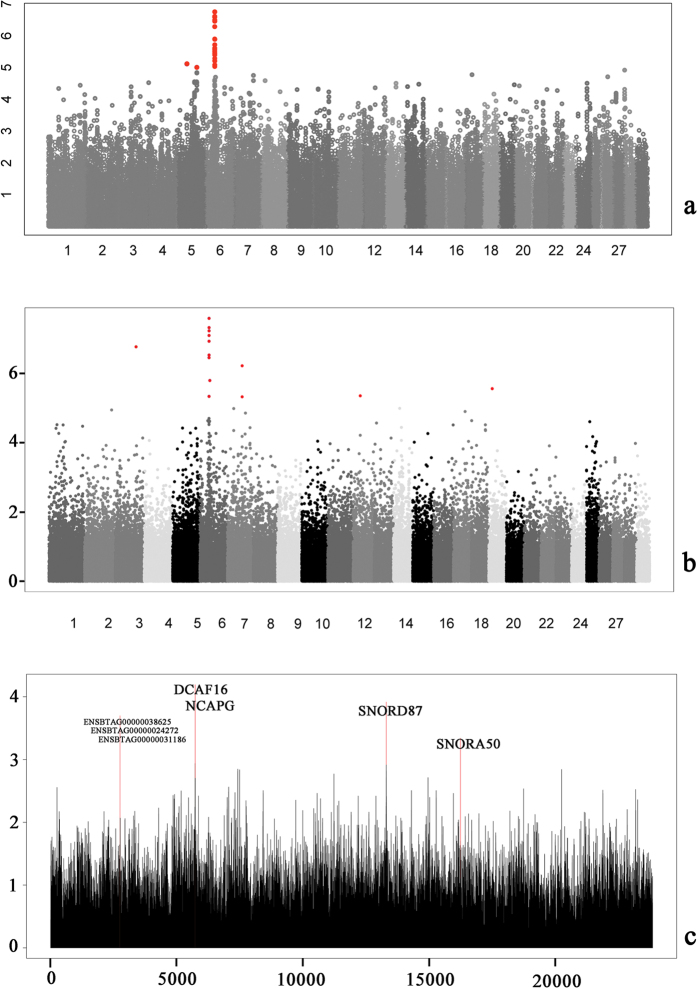
Results of the multi-strategy GWAS for average daily gain. (**a**) Manhattan plots for the SNP-based GWAS. (**b**) Manhattan plots for the haplotype-based GWAS. (**c**) Log10 (P-value) values of 23,856 genes in the gene-based GWAS.

**Figure 2:**
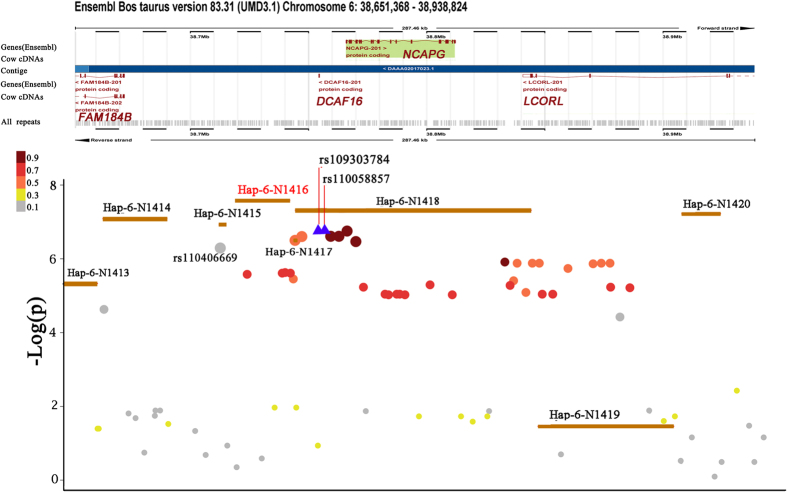
Regional −Log10 (P-value) plot of the SNP-based and haplotype-based association around the *DCAF16-NCAPG* locus on BTA6: 38.6–39.0(Mb). The yellow bar represents the block position. The purple triangle represents the two most significant SNPs (rs109303784 and rs110058857, r^2^ = 1). SNPs were colored based on their LDs with two most significant SNPs as follows: red SNPs with LDs at r^2^ > 0.9, pink SNPs with LDs at r^2^ > 0.7, orange SNPs with LDs at r^2^ > 0.5, yellow SNPs with LDs at r^2^ > 0.3 and grey SNPs with LDs at r^2^ < 0.3. The size of the plots indicates the significance level of SNPs in the SNP-based GWAS. The positions of all RefSeq genes were downloaded from the ENSEMBL database.

In this study, we used three strategies to perform a genome-wide association study (GWAS) for the ADG trait in beef cattle (Fig. 1). In the SNP-based association, we identified 40 distinct SNPs (Supplementary Table S2) that exceeded the suggested significance thresholds (P < 10^−6^), 38 of which were located within BTA6 (Fig. 1a). Here, we identified the most significant SNPs, rs109303784 and rs110058857, on BTA6 with identical P-values of 1.78 × 10^−7^. The distance between the two significant SNPs was 680 bp, which were in complete linkage disequilibrium (r^2^ = 1) and explained 4.01% of the phenotypic variance. Rs109303784 and rs110058857 were both located upstream of *NCAPG* and downstream of the *DCAF16* gene according to the Ensembl genome database (http://www.ensembl.org/index.html). Figure 2 showed the regional −Log10 (P-value) of the significant SNPs that surround the *DCAF16-NCAPG* locus on BTA6. We also calculated the LD levels, with the two peak SNPs denoted by different colors. Notably, we found that rs110406669 (P = 5.18 × 10^−6^) had a low LD with the two peak SNPs and independently explained 3.32% of the phenotypic variance. Moreover, two other prominent SNPs, rs109028700 (BTA5:43111315) and rs137683327 (BTA5:84944556), were located on BTA5 and explained 2.59% and 2.87% of the phenotypic variance, respectively.

### Haplotype-based GWAS results

A total of 93,732 blocks were identified, and these blocks comprised 615,355 SNPs. The maximum length was 99.9 Kb, and the minimum length was 0.4 Kb. Fourteen significant haplotype blocks (shown in Table 2) were obtained at the suggested threshold (P < 10^−5^) across 5 chromosomes (BTA3, BTA6, BTA7, BTA12, and BTA19). Similar to the SNP-based GWAS, 7 associated haplotype blocks that surrounded rs109303784 and rs110058857 were found on BTA6 (Fig. 2). The most significant block, Hap-6-1416 (P = 2.56 × 10^−8^), spanned 22.8 Kb and was located in upstream of *NCAPG* at a distance of 12.7 Kb with rs109303784. The Hap-6-N1416 block explained 4.85% of the phenotypic variance and had 7 distinct haplotypes (GTGGATA, GTGAATA, GTAAATA, ACAGGCG, ACAAGCG, ACAAATA and ATAAATA, referred to as Haplo1, Haplo2, Haplo3, Haplo4, Haplo5, Haplo6 and Haplo7) with frequencies of 0.13%, 2.67%, 4.94%, 19.36%, 5.74%, 1.34% and 65.82%, respectively. The average effect was 0.24 kg/day, with the minimum in Haplo3 of 0.08 kg/day and the maximum in Haplo5 of 0.45 kg/day.

In contrast to the SNP-based GWAS results, no prominent block was found on BTA5, but 5 blocks were identified on BTA3, 7, 12 and 19. However, no gene regions or coding domains coincided with these blocks. Notably, Hap-3-N3218 (P = 1.7 × 10^−7^) on BTA3 contained 3 extragenic SNPs (rs109934393, rs43349539 and rs43348574) that explained 6.22% of phenotypic variance. These results indicated that unknown functional regions or regulatory elements may exist around this identified block.

### Gene-based GWAS results

A total of 24,616 genes were annotated in ENSEMBLE database. For the gene-based association, 23,856 genes with an average 34.7 SNPs per gene were analyzed. And other 760 genes were excluded, since they included less than three SNPs or not were located in autosomes (sex chromosome or mitochondria DNA). The 1,000 permutation-cycle results suggested a set P-value of 10^−3^ with a FDR < 1%. Seven genes were identified for ADG in this study (Table 3). Specifically, *DCAF16* and *NCAPG* were implicated by the SNP- and Haplotype-based association results. We also found two small nucleolar RNAs, SNORD50 and SNORD87, with identical functions in the modification process of other small nuclear RNAs (snRNAs). Additionally, two uncharacterized proteins—ENSBTAG00000038625 and ENSBTAG00000024272—were obtained. These results indicated that the gene-based method can identify functional genes or loci which are previously unverified and provide a possible structural basis for further gene functional validation studies.

### *DCAF16-NCAPG* locus associated with ADG

**Figure 3:**
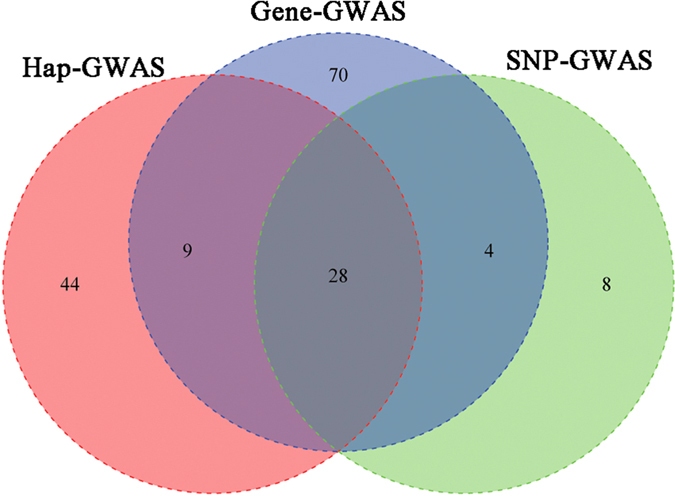
Venn diagram summarizing the association analyses results of the three strategies. The number represents the interaction and the remaining significant SNPs identified in three GWAS methods.

Taken together, 163 significant SNPs were identified by three GWAS strategies (The SNPs in the gene-based set were SNPs within significant genes). Venn diagram summarizing the three strategies results was shown in Fig. 3. Here, the SNP- and haplotype-based GWAS approaches returned a distinct set of 8 and 44 prominent SNPs, respectively. Five genes—*PTPRR, LMNTD1, FAM114A2, C8A* and *STARD13*—were proximal to these 52 significant SNPs, suggesting associations for some of these genes with the ADG trait. We focused on the intersection of candidate SNPs identified by the three GWASs methods with the highest ADG trait-associated accuracy, which included 28 significant SNPs located at 38.6–39.0 Mb on BTA6. Figure 2 showed a schematic diagram of the region, which contains four annotated genes—*FAM184B, DCAF16, NCAPG*, and *LCORL*—from the Ensembl genome database.

**Figure 4:**
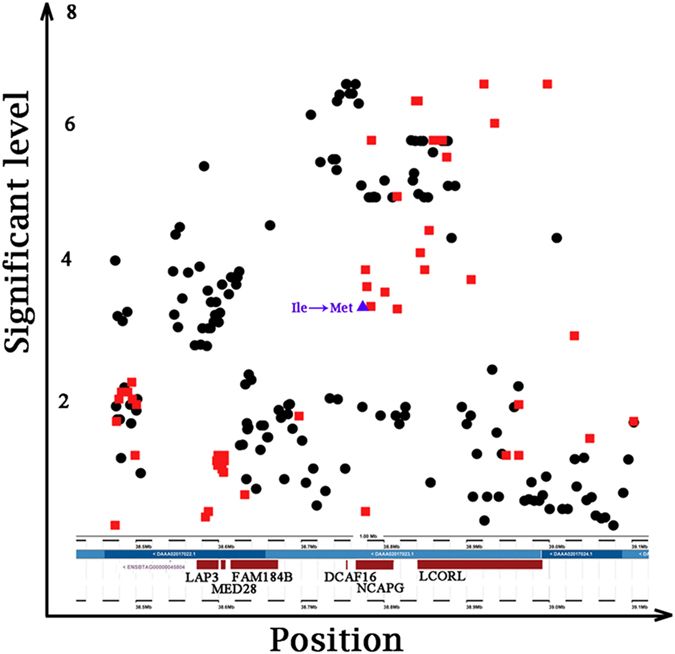
Regional plot of our GWAS results versus association analysis results by Lindholm-Perry^40^ . The black circles represent the −Log10 (P-value) of our SNP-based GWAS, and the red squares represent the −Log10 (P-value) of the previous association analysis. The purple triangle represents SNP c.1326 T > G, which is the Ile442 to Met442 amino acid change, in exon 9 of *NCAPG*.

*DCAF16*, which was near to the peak SNPs for SNP-based GWAS approach, was the most significant gene (P = 6.45 × 10^−5^) for gene-based GWAS analysis. Similarly, the most significant block, Hap-6-N1416 (P = 2.56 × 10^−8^), was also located downstream of *DCAF16* (physical distance = 19,663 bp) according to the Ensembl database. *DCAF16* may function as a substrate receptor for the *CUL4-DDB1 E3* ubiquitin-protein ligase complex, which is involved in two pathways that promote protein modifications and ubiquitination. *NCAPG*, which was also identified by three GWAS methods simultaneously, encodes a subunit of the condensin complex, which is responsible for the condensation and stabilization of chromosomes during mitosis and meiosis. The associated pathways involved the cell cycle, mitosis and the mitotic prometaphase. Numerous studies^25^,^26^,^40^,^41^,^42^,^43^,^44^,^45^,^46^,^47^,^48^,^49^,^50^,^51^,^52^ have confirmed that *NCAPG* has strong effects on the body sizes and growth traits of human and domestic animals. According to the association analyses from Lindholm-Perry’s results^40^, 47 SNPs within or near the gene boundaries of the three candidate genes (*NCAPG, LCORL* and *LAP3*) were genotyped. Figure 4 showed a comparison of these association study results with our SNP-based GWAS results. In contrast to our results, the most significant SNPs were located in the *LCORL* gene. However, most of the significant SNPs from these two analyses were located around the BTA6: 38.78 (Mb) region near the downstream region of *DCAF16*, suggesting that this region might be a more effective QTL for ADG trait in cattle.

Additionally, a missense mutation (c.1326 T > G, indicated in Fig. 4 by a purple triangle) was identified in exon 9 of *NCAPG* by several association^26^,^45^ and linkage analyses^25^,^41^. The resulting amino acid change of Ile442 to Met442 in the encoded protein has been shown to be a candidate causative variation of the growth trait in cattle. Significant selection regions that affect the statures of European and African cattle cohorts were identified in *NCAPG* by multiple signal selection analyses^49^. GWAS analyses in horses^42^,^43^,^46^,^47^ and cattle^48^,^51^ indicated that the *NCAPG-LCORL* locus or closed regions were significantly associated with body size and growth traits.

Based on our results and previous reports^44^,^52^, we tested *DCAF16, NCAPG*, and *LCORL* expression in muscle tissues. Longissimus dorsi muscle samples from 28 steers with ADG phenotypes were collected. General linear model (GLM) results showed *DCAF16* and *NCAPG* expression is significantly associated with ADG trait (Table 4) and correlations between ADG and genes expression were presented in (Supplementary Fig. S3). No significant difference was detected for the *LCORL* gene. Our results were concordant with the results presented by Perry *et al*.^44^ that abundance of *NCAPG* was associated with ADG in the muscle tissue muscle from cows.

In the NCBI database, the *NCAPG* gene has one reference transcript (Genebank accession number: NM_001102376) and two predicted transcripts (XM_005207785 and XM_015471561), which were derived by a computational analysis using transcriptome data from 11 Hereford cattle. The differences between the three transcripts occur in exon 1 (Supplementary Fig. S4). Three transcripts primers were designed using the Primer 5 software (Supplementary Table S3). We demonstrated the existence of three transcripts in Simmental cattle using reverse transcription polymerase chain reaction (RT-PCR) (Supplementary Fig. S2), and the PCR production sequences were consistent with those reported in the NCBI database. To address the significant association between each transcripts abundance and the ADG trait, we also tested the expression levels of three transcripts. GLM results showed XM_005207785 (P = 0.050) expression was significantly associated with ADG, while no significant correlation were found in NM_001102376 (P = 0.597) and XM_015471561 (P = 0.074) transcripts (Table 4).

Overall, *DCAF16* and *NCAPG* have been simultaneously explored by the three GWAS methods, and statistical analysis have proven that *DCAF16* and one of *NCAPG* transcripts (XM_005207785) abundance were associated with ADG trait, indicating that the *DCAF16*-*NCAPG* region is a susceptibility locus for the ADG trait in cattle.

Furthermore, we noticed that the independent and significant SNP (rs110406669) from the SNP-based GWAS was located 5′ upstream with a distance of 30,695 bp to XM_005207785. Two peak SNPs were located in intron 1 of XM_005207785 and upstream with a distance of 6,970/7,650 bp to DCAF16. We then searched the transcription factor-binding (TF) site around candidate regions using the Tfsitescan software on the MIRAGE WWW server (http://www.ifti.org/cgi-bin/ifti/Tfsitescan.pl). The regions, which contained ±5 Kb flanking sequences of the obtained significant SNPs (rs109303784, rs110058857 and rs110406669), were analyzed, and Table 5 showed the Tfsitescan results. The distances between the two most significant TF sites identified here—Nmp4-COL1A1-sit and AT2-VIRE—and the significant SNPs were 130 bp and 178 bp, respectively. It has been shown that Nmp4-COL1A1-sit influences cell structure and function during extracellular matrix remodeling in osteoblasts^53^. The protein product of AT2-VIRE, the AT2 receptor, is widely and abundantly expressed in fetal tissues and plays a pivotal role in cell differentiation and growth^31^. Moreover, similar TF site sequences were found upstream of the NCAPG gene in various species (Supplementary Table S4). Taken together, we proposed that Nmp4-COL1A1-sit, AT2-VIRE or other TF sites are probably involved in the regulation of DCAF16 or NCAPG transcript expression in association with the ADG trait.

## Conclusion

In this study, we performed multi-strategy GWASs to investigate average daily gain (ADG) in the Simmental beef cattle. Forty significant SNPs in the SNP-based GWAS, 14 significant haplotype blocks in the haplotype-based GWAS, and 7 prominent genes in the gene-based GWAS were identified. Two genes, *DCAF16* and *NCAPG*, were demonstrated to be associated with ADG by all three GWAS methods. Most importantly, the significant SNPs within the *NCAPG*-*DCAF16* region were strongly associated with the ADG trait, with phenotypic variance of approximately 4%, suggesting the existence of causal variants in this region. Moreover, we have also shown that *DCAF16* and *NCAPG* expression were significantly associated with ADG. Our findings provide insights into the understanding of the genetic mechanisms underlying ADG trait in cattle, and these results inform future NGS-GWAS analyses of causal variants for the ADG trait. Moreover, multi-strategy GWASs represents a powerful approach to the search and analysis of susceptibility loci-related traits.

## Additional Information

**How to cite this article**: Zhang, W. *et al*. Multi-strategy genome-wide association studies identify the DCAF16-NCAPG region as a susceptibility locus for average daily gain in cattle. *Sci. Rep.* **6**, 38073; doi: 10.1038/srep38073 (2016).

**Publisher's note:** Springer Nature remains neutral with regard to jurisdictional claims in published maps and institutional affiliations.

## Supplementary Material

Supplementary Files

## Figures and Tables

**Table 1:** Average daily gain (ADG)-associated quantitative trait loci (QTL) in cattle.

Years	Breeds	Method^1^	Position (Mbp)^2^	Candidate genes^3^	Reference
2004	M1 line^4^	QTL	2: 6.5–14.46: 6.6–8.5		54
2007	Angus, Charolais,Alberta Hybrid crosses	QTL	5: 71.4–71.66:10.9–11.1**6: 29.0–29.6**7: 44.2–46.9		55
2008	Continental × British^5^	Association	29: 38.1–39.9	*IGF2*	56
2011	Crossbred	Association	**6: 21.0–22.0**	***NCAPG***	40
2011	Wagyu	Association	1: 60.9–61.16: 4.9–9.3	*GHSR*	57
2012	Angus	GWAS	**6: 27.0–29.1**		58
2012	M1 line	QTL	5: 32.0–34.2		59
2012	Brangus heifers	GWAS	**6: 31.7–32.4**10: 33.1–33.5		60
2013	Angus, Chaorlais, Piedmontese	GWAS	1: 51.3–51.4		61
2013	Angus × Simmental crossbred	GWAS	2: 11.3–11.615: 11.3–11.6	*NCKAP5,PHOX2 A*	62
2013	Nanyang^6^	Association	7: 24.3–24.5	*PROP1*	63
2015	Nanyang	Association	22:16.9–16.9	*CIDEC*	64

Note: ^1^“GWAS” refers to the genome-wide association study, “Association” refers to the candidate gene association analysis, and “QTL” refers to the QTL mapping linkage association analysis. ^2^QTL positions that were reported as associated with the ADG trait. ^3^Candidate genes that were reported as associated with the ADG trait. ^4^The M1 line of Beefbooster Inc. was developed from an Angus base and has been under selection for over 30 years. ^5^Samples were hybrid beef steers sired by Angus, Charolais, or University of Alberta Hybrid bulls. Dams were from three composite lines. ^6^Nanyang is a breed of Chinese native cattle. The QTL regions that are reported to surround the *DCAF16-NCAPG* locus are indicated in Boldface.

**Table 2:** Significant haplotypes from the haplotype-based GWAS.

Hap.^1^	SNP	Start	End	Len.^2^	Chr^3^	P-value	Effect^4^
Hap-6-N1416	rs137684828 rs133288426 rs136487046 rs110302064 rs109776587 rs110281825	38711873	38734752	22,879	6	2.56 × 10^−8^	4.85%
Hap-6-N1418	27^5^	38737206	38837159	99,953	6	4.75 × 10^−8^	4.60%
Hap-6-N1420	rs132741545 rs137111419	38900275	38917456	17,181	6	5.88 × 10^−8^	4.64%
Hap-6-N1414	rs136542559 rs110770764 rs110867784 rs110780166 rs109035277 rs134219500 rs109920396	38655605	38682695	27,090	6	8.02 × 10^−8^	4.38%
Hap-6-N1415	rs110406669 rs134225464	38704872	38707716	2,844	6	1.18 × 10^−7^	4.98%
Hap-3-N3218	rs109934393 rs43349539 rs43348574	89890121	89893239	3,118	3	1.70 × 10^−7^	6.22%
Hap-6-N1417	rs109448564 rs109436638	38735901	38736441	540	6	2.95 × 10^−7^	3.59%
Hap-6-N1409	rs110983998 rs109705804 rs110638909 rs110338374 rs110878984 rs109627413 rs136745840	38477781	38495361	15,411	6	3.50 × 10^−7^	3.87%
Hap-7-N2309	rs137459122 rs136096935 rs136896599	67220287	67227674	7,387	7	6.02 × 10^−7^	3.73%
Hap-6-N1536	rs43457333 rs43457339 rs43457349 rs43457352 rs43457353 rs43455999	42130983	42147873	16890	6	1.60 × 10^−6^	2.00%
Hap-19-N540	rs110686148 rs135073323	13534451	13535938	1,487	19	2.68 × 10^−6^	3.26%
Hap-12-1130	rs137645749 rs41600431 rs137228197 rs135990217 rs29021098	28042816	28052265	9,449	12	4.42 × 10^−6^	2.79%
Hap-6-N1413	rs109849093 rs110793327 rs109063701 rs109622396	38644886	38653201	8,315	6	4.59 × 10^−6^	3.16%
Hap-7-N2310	rs133024924 rs134650380 rs135673337 rs132656697 rs134402892	67237316	67243709	6,393	7	4.74 × 10^−6^	2.52%

Note: ^1^Haplotype name. ^2^Length (bp). ^3^Chromosome. ^4^Phenotypic variance explained by the haplotype. ^5^Twenty-seven SNPs were listed as follows: rs135282158 rs137844992 rs38746212 rs109303784 rs110058857 rs110412527 rs109414269 rs110841247 rs109885206 rs109554838 rs110443327 rs109322908 rs109861428 rs110062110 rs109002201 rs110024295 rs133222819 rs137268410 rs109801011 rs110426625 rs135260513 rs109795992 rs110030099 rs110908263 rs110386170 rs110419157 rs109924798.

**Table 3:** Seven significant ADG-associated genes based on the gene-based GWAS.

ENSEMBL ID	Description	P-value	FDR	Number of SNPs	Chr
ENSBTAG00000011973	DDB1 and CUL4 associated factor 16 (*DCAF16*)	6.45 × 10-5	0.0016	30	6
ENSBTAG00000021582	non-SMC condensin I complex, subunit G (*NCAPG*)	1.12 × 10-4	0.0043	42	6
ENSBTAG00000043234	Small nucleolar RNA SNORD87	1.22 × 10-4	0.0045	17	14
ENSBTAG00000038625	Uncharacterized protein	2.01 × 10-4	0.0045	3	3
ENSBTAG00000024272	Uncharacterized protein	2.01 × 10-4	0.0049	4	3
ENSBTAG00000031186	Guanylate binding protein family, member 6 (*GBP6*)	2.11 × 10-4	0.0057	4	3
ENSBTAG00000043192	Small nucleolar RNA SNORA50	4.57 × 10-4	0.0084	44	18

**Table 4:** Target gene expression in muscle tissue and estimated effects for ADG.

Gene	Effect (SE)^1^	*P*-value
***NCAPG***	**0.018 (±0.009)**	**0.046**
*LCORL*	0.050 (±0.032)	0.138
***DCAF16***	**0.030 (±0.012)**	**0.046**
NM_001102376	0.003 (±0.006)	0.597
**XM_005207785**	**0.012 (±0.006)**	**0.050**
XM_015471561	0.007 (±0.004)	0.074

Note: ^1^Estimate effect in kg/day and standard error of unit of transcript abundance in fold change of genes on ADG.

**Table 5:** List of transcription factor-binding (TF) sites around the *NCAPG-LCORL* locus.

Significant SNP	Site (Length)	Position^1^ (bp)	Occurrence	Exp. Value^2^	Significance Level^3^	Distance^4^ (bp)
rs110406669	Zp2-Ebox (14)	−34345	1	3.81 × 10^−4^	*	3627
	Fkh/HNF-3-subcl’ (15)	−31870	1	2.82 × 10^−4^	*	1152
	**Nmp4-COL1A1-sit’ (20)**	**−30848**	**1**	**1.31 × 10**^−7^	***	**130**
	flk1-HBE’ (14)	−29997	1	3.81 × 10^−4^	*	721
	CTF/NF1-CF1-2 (11)	−27444	1	5.67 × 10^−4^	*	3274
	delta-globin-PY (10)/pyr factor site (10)	−25949	1	7.50 × 10^−4^	*	4769
	gamma-globin-he’ (13)	−25836	1	4.65 × 10^−4^	*	4882
rs110058857 and rs110058857	Sp1-U2snR.2 (11)	+6963	1	5.67 × 10^−4^	*	4892
	PRDI_(1)’ (14)	+7799	1	3.81 × 10^−4^	*	4056
	Elf-1-FCER1A’ (11)	+7828	1	5.67 × 10^−4^	*	4027
	mTH-Pitx3-site- (14)	+9508	1	3.81 × 10^−4^	*	2347
	CBF-CYP2E1 (14)	+10839	1	3.81 × 10^−4^	*	1016
	**AT2-VIRE (16)**	**+11677**	**1**	**2.71 × 10**^**−5**^	***	**178**
	C/EBP-IL-8-1 (14)	+11954	1	3.81 × 10^−4^	*	99
	IgHC.11′ (12)	+14748	1	1.42 × 10^−4^	*	2893
	SV40.10(11)/SV40.4(11)	+15055	2	5.67 × 10^−4^	*	3200

Note: ^1^“Position” indicates distance (in nucleotides) relative to the beginning of XM_005207785; negative indicates upstream of XM_015471561. ^2^Experience Value. ^3^Significance level is represented by an asterisk (*), where *indicates 10^−4^ < P-Value < 10^−3^, and ***indicates P-Value < 10^−4^. ^4^Distance between TF and significant SNPs.
