# Supplementary material for: Multi-strategy genome-wide association studies identify the DCAF16-NCAPG region as a susceptibility locus for average daily gain in cattle
Source: Sci Rep. 2016 Nov 28;6:38073. doi: 10.1038/srep38073 (PMC5125095; doi:10.1038/srep38073)
Supplement: Supplementary Files [file srep38073-s1.pdf]

Multi-strategy genome-wide association studies identify the DCAF16-NCAPG region as a susceptibility locus for average daily gain in cattle

Wengang Zhang[1], Junya Li[1], Yong Guo[2], Lupei Zhang[1], Lingyang Xu[1], Xue Gao[1], Bo Zhu[1], Huijiang Gao[1], Hemin Ni[2*], & Yan Chen[1*]

[1]Institute of Animal Science, Chinese Academy of Agricultural Science, Beijing 100193, China

[2]Animal Science and Technology College, Beijing University of Agriculture (BUA), Beijing 102206, China.

***Corresponding authors:**

Hemin Ni	Email: nihemin@aliyun.com
Yan Chen	Email: chenyan0204@163.com

Email address of other authors:

Wengang Zhang	Email: zhangwengang_19@sina.com
JunYaLi	Email: JL1@iascaas.net.cn
Yong Guo	Email: y63guo@126.com
Lupei Zhang	Email: lpzhang@iascaas.net.cn
Lingyang Xu	Email:xulingyang@163.com
Bo Zhu	Email: zhubo525@126.com
Xue Gao	Email: gaoxue76@126.com
Huijiang Gao	Email: gaohj111@sina.com

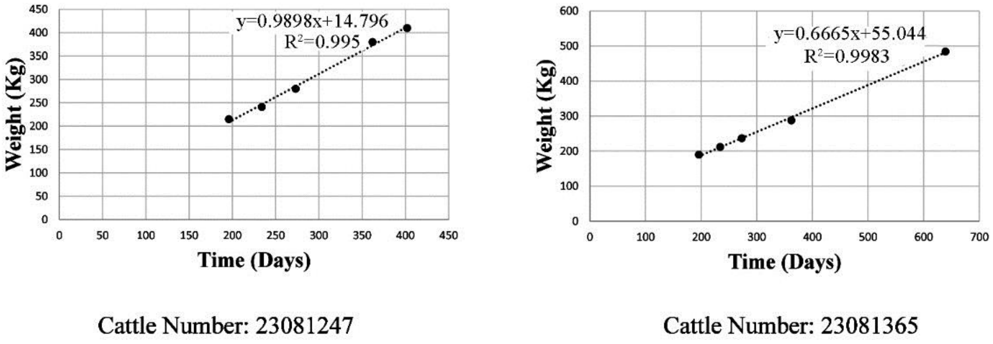

Supplementary Figure S1. Growth curve during the fattening period.

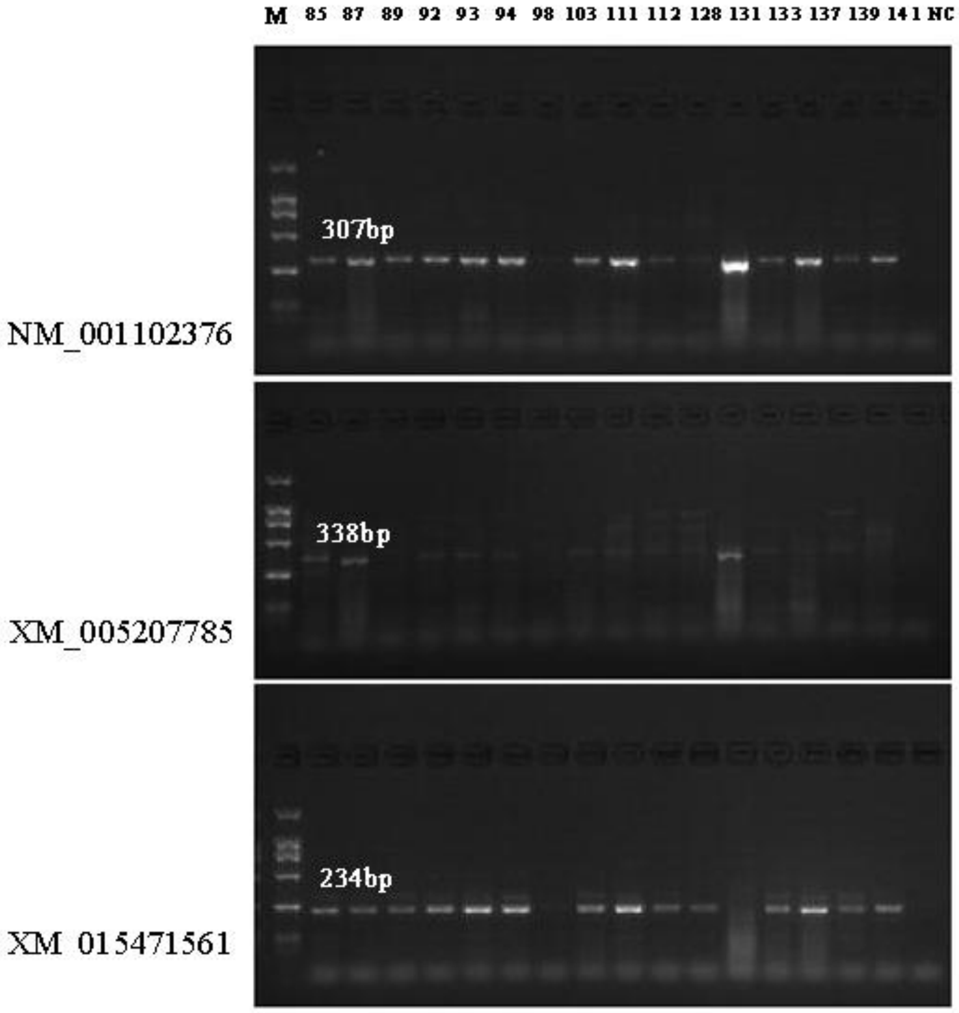

Supplementary Figure S2. PCR production of three transcripts in exon 1 of the *NCAPG* gene. Sixteen longissimus muscle cDNA samples were amplified. M: DL2000 Marker, NC: negative control.

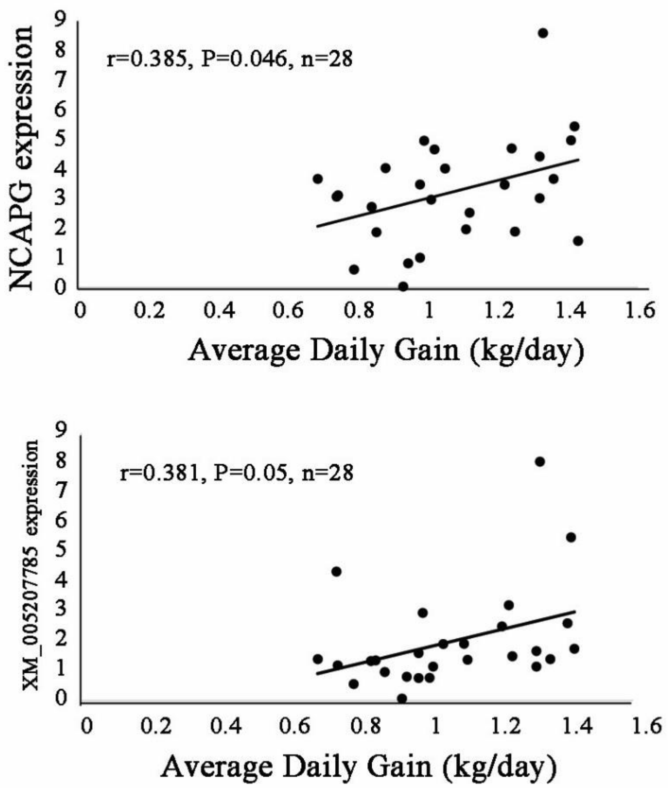

Supplementary Figure S3. Correlation between candidate genes expression and average daily gain trait in 28 steers.

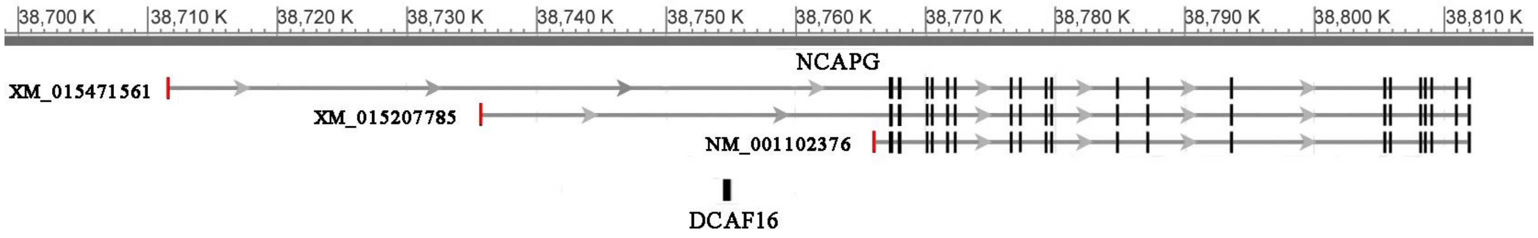

Supplementary Figure S4. Gene structures of the NCAPG and DCAF16 genes. Black boxes represent the coding sequences, and the red boxes represent exon 1 in each transcript.

Supplementary Table S1. Primer sequences used for qRT-PCR

Gene name	Genebank ID	F Primer (5'-3')	R Primer (5'-3')	Length	Exons[1]
NCAPG	NM_001102376	GTAGGCGAACGTGAACAG	CATCATTTTCCATATCTGATTGGT	307	1, 2
	XM_005207785	AGAATTGCCGGAGATTTACC	TCGTTTATAGACCACCATAGC	154	1, 2
	XM_015471561	AAATGTGACAGGTGCAATTTTTTCC	CATCATTTTCCATATCTGATTGGT	237	1, 2
	FULL	CTTTCCGATTTCTTAGATAGTGAG	AAACATTCCTGGTTAGTCCTG	224	14, 15
DCAF16	NM_001078147	GCCTCCTCTGGCTTCTTG	GTGGGGGGTGGCTCTACT	148	
LCORL	NM_001192357	GTGAACCAGAAGAGCTGACTGA	GTTCCTCTGTTGGTGTTGACTG	126	1, 2
GAPDH	NM_001034034	ATCATCTCTGCACCTTCTGCCGAT	TAAGTCCCTCCACGATGCCAAAGT	166	3, 4

Note: [1]The exons were the primers.

Supplementary Table S2. Significant SNPs based on the SNP-based GWAS

SNP	Chromosome	Position	P-value	MAF	Effect
**BovineHD0600010730(rs109303784)**	**6**	**38747445**	**1.78E-07**	**0.270216**	**4.01%**
**BovineHD0600010731(rs110058857)**	**6**	**38748125**	**1.78E-07**	**0.270216**	**4.01%**
BovineHD0600010735	6	38758660	1.78E-07	0.270216	3.76%
BovineHD0600010733	6	38751637	2.47E-07	0.270889	3.62%
BovineHD0600010734	6	38754991	2.47E-07	0.270889	3.62%
BovineHD0600010729	6	38739408	2.57E-07	0.315364	3.7%
BovineHD0600010727	6	38736441	3.23E-07	0.316712	3.33%
BovineHD0600010736	6	38762470	3.50E-07	0.277628	3.41%
**BovineHD0600010716(rs110406669)**	**6**	**38704872**	**5.18E-07**	**0.327493**	**3.32%**
Hapmap27083-BTC-041166	6	38825835	1.27E-06	0.278976	3.60%
BovineHD4100004575	6	38830725	1.30E-06	0.320081	3.20%
BovineHD4100004577	6	38837159	1.30E-06	0.320081	3.20%
BovineHD4100004578	6	38840174	1.30E-06	0.320081	3.20%
BovineHD4100004581	6	38862997	1.30E-06	0.320081	3.20%
BovineHD0600010755	6	38866381	1.30E-06	0.320081	3.20%
Hapmap31285-BTC-041097	6	38869785	1.30E-06	0.320081	3.20%
BovineHD4100004580	6	38852093	1.93E-06	0.341644	2.99%
BovineHD0600010723	6	38731368	2.46E-06	0.252022	3.00%
BovineHD0600010725	6	38732667	2.46E-06	0.252022	2.98%
BovineHD4100004573	6	38734752	2.46E-06	0.252022	3.32%
BovineHD0600010719	6	38716298	2.71E-06	0.264825	2.88%
Hapmap26308-BTC-057761	6	38576012	3.13E-06	0.358491	3.19%
BovineHD0600010726	6	38735901	3.58E-06	0.29717	3.00%
BovineHD0600010752	6	38829248	4.00E-06	0.338949	3.29%
BovineHD0600010745	6	38793698	5.18E-06	0.250674	3.02%
BovineHD0600010751	6	38827869	5.18E-06	0.250674	3.01%
BovineHD0600010737	6	38765656	6.15E-06	0.260108	2.75%
BovineHD4100004582	6	38870271	6.24E-06	0.25	2.65%
BovineHD0600010757	6	38878824	6.24E-06	0.25	2.81%
BovineHD0500012336	5	43111315	7.71E-06	0.416442	2.88%
BovineHD4100004576	6	38834676	8.29E-06	0.292453	2.77%
BovineHD0600010742	6	38781062	9.11E-06	0.256065	2.50%
BovineHD0600010739	6	38774879	9.30E-06	0.258086	2.44%
BovineHD0600010740	6	38776500	9.30E-06	0.258086	2.44%
BovineHD0600010741	6	38779440	9.30E-06	0.258086	2.44%
BovineHD0600010743	6	38783076	9.30E-06	0.258086	2.44%
BovineHD0600010746	6	38803310	9.30E-06	0.258086	2.44%
BovineHD4100004579	6	38841588	9.30E-06	0.258086	2.44%
Hapmap23507-BTC-041133	6	38845992	9.30E-06	0.258086	2.44%
BovineHD0500024028	5	84944556	9.83E-06	0.398248	2.51%

Supplementary Table S3. Primer sequences and reaction conditions for PCR amplification.

Primer	Primer sequences (5′-3′)	Annealing temperature (°C)	Fragment size (bp)	Transcripts
P1	F:gtaggcgaacgtgaacag<br>R:catcattttccatatctgattggt	56	307	NM_001102376
P2	F:cgcaaggagatggcg<br>R:catcattttccatatctgattggt	56	338	XM_005207785
P3	F:aaatgtgacaggtgcaattttttcc<br>R:catcattttccatatctgattggt	56	234	XM_015471561

Supplementary Table S4. Transcription factor BLAST results

Species	**Nmp4-COL1A1-sit'**		**AT2-VIRE**	
	Position	P-value	Position	P-value
Homo sapiens	Chr4: 17774992	7e-6		
	17748126	3e-5		
	17754569	3e-5		
	17802658	3e-5		
Sus scrofa	Chr8: 12532242	7e-6		
	12505074	3e-5		
	12517099	3e-5		
	12528671	3e-5		
Ovis aries	Chr6: 37233525	4e-5	Chr6: 37271938	1e-04
Equus caballus	Chr3: 105847261	5e-5		
	105841839	5e-5		
	105897833	5e-5		
	105900737	5e-5		
